# Genetic Analysis of Benzothiophene Biodesulfurization Pathway of *Gordonia terrae* Strain C-6

**DOI:** 10.1371/journal.pone.0084386

**Published:** 2013-12-19

**Authors:** Wei Wang, Ting Ma, Kehui Lian, Yue Zhang, Huimei Tian, Kaihua Ji, Guoqiang Li

**Affiliations:** 1 Key Laboratory of Molecular Microbiology and Technology, Ministry of Education, College of Life Sciences, Nankai University, Tianjin, P. R. China; 2 TEDA School of Biological Sciences and Biotechnology, Nankai University, Tianjin, P. R. China; 3 Tianjin Key Laboratory of Microbial Functional Genomics, Tianjin, P. R. China; Universidade Nova de Lisboa, Portugal

## Abstract

Sulfur can be removed from benzothiophene (BT) by some bacteria without breaking carbon-carbon bonds. However, a clear mechanism for BT desulfurization and its genetic components have not been reported in literatures so far. In this study, we used comparative transcriptomics to study differential expression of genes in *Gordonia terrae* C-6 cultured with BT or sodium sulfate as the sole source of sulfur. We found that 135 genes were up-regulated with BT relative to sodium sulfate as the sole sulfur source. Many of these genes encode flavin-dependent monooxygenases, alkane sulfonate monooxygenases and desulfinase, which perform similar functions to those involved in the 4S pathway of dibenzothiophene (DBT) biodesulfurization. Three of the genes were found to be located in the same operon, designated *bds*ABC. Cell extracts of pET28a-*bds*ABC transfected *E. coli* Rosetta (DE3) converted BT to a phenolic compound, identified as o-hydroxystyrene. These results advance our understanding of enzymes involved in the BT biodesulfurization pathway.

## Introduction

Sulfur-oxide gases (mostly SO_2_) are released during the combustion of sulfur compounds, which results not only in serious air pollution, but also poison metal catalysts [1]. Benzothiophene (BT), dibenzothiophene (DBT), and their alkylated derivatives, account for more than 50% of the total sulfur content of commercial diesel [2,3]. These aromatic thiophenes are recalcitrant organic sulfur compounds and more resistant to hydrodesulfurization (HDS) treatment than other sulfur compounds such as mercaptans and sulfides [4]. Fortunately, several bacterial species can efficiently desulfurize aromatic thiophenes such as BT, DBT and their derivatives under ambient temperature and pressure [5].

As DBT is a common organosulfur compound found in a variety of fuels and more resistant to HDS treatment than other thiophene sulfides, it is widely regarded as a model compound for the isolation and characterization of biodesulfurization bacteria [6]. Two major pathways of DBT biodesulfurization have been reported and designated the “Kodama” and “4S” pathways, respectively. In the former, DBT is converted to hydrophilic organosulfur compounds by a series of oxidations of one of the aromatic rings of DBT [7]. The latter is a non-destructive desulfurization pathway and has been widely studied. In the “4S” pathway, DBT is successively converted to sulfoxide (DBTO), sulfone (DBTO_2_), sulfinate (HPBSi) and hydroxybiphenyl (HBP) without degradation of either aromatic ring [8]. These four steps catalytic reactions are performed by three enzymes (DszC, DszA, and DszB), which are encoded by a single operon (*dsz*ABC). DBT is initially oxidized by DszC, first to DBT-5-oxide (DBTO) and then to DBT-5, 5'-oxide (DBTO_2_). DszA catalyzes the transformation of DBTO_2_ to 2-(2'-hydroxyphenyl) benzene sulfinate (HPBSi), which opens the thiophenic ring. HPBSi is then desulfinated by DszB to produce 2-hydroxybiphenyl (2-HBP) [9]. These enzymes have been isolated, cloned, mutated, overexpressed, and crystallized [10-14]. It was found that DszC and DszA are flavin-dependent monooxygenases that require FMNH_2_ or FADH_2_ for their activities. Therefore, an additional enzyme, flavin reductase, is required, which catalyzes the reduction of flavins, such as FMN and FAD, by NAD(P)H to form reduced flavins.

**Figure 1:**
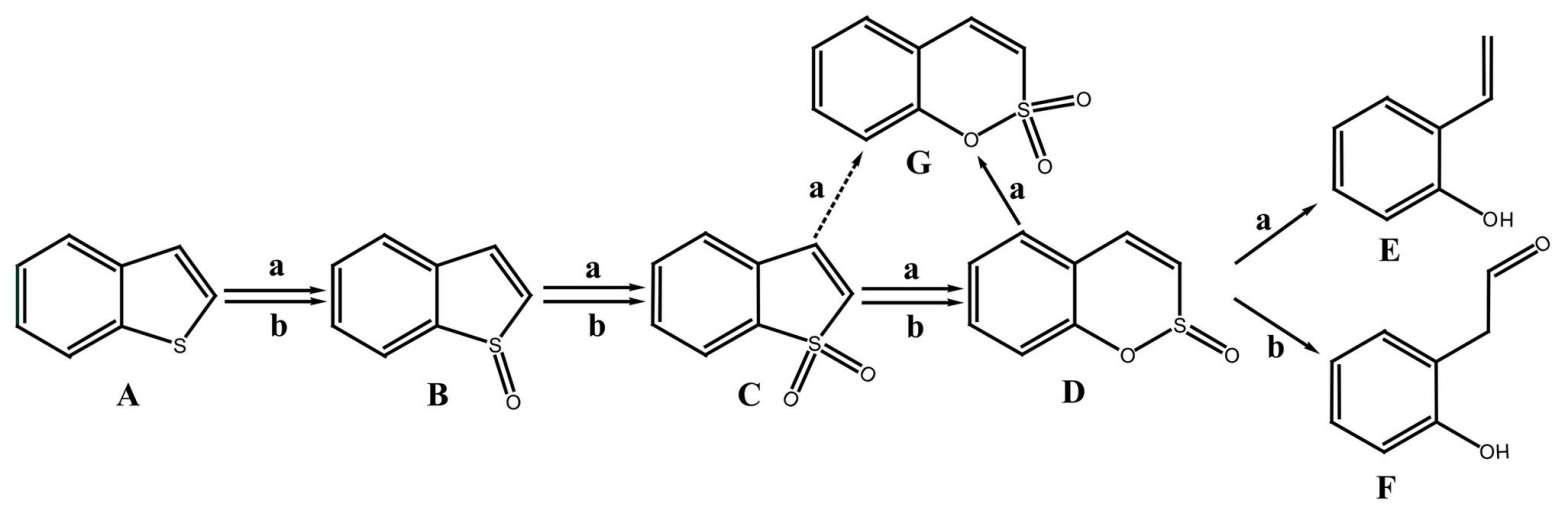
The proposed BT biodesulfurization pathway for *Sinorhizobium* sp. KT55 (a) and *Gordonia* sp. 213E (b). (A) benzothiophene; (B) benzothiophene S-oxide; (C) benzothiophene S,S-dioxide; (D) benzo[c][l,2]oxathiin S-oxide; (E) o-hydroxystyrene; (F) 2-(2’-hydroxypheny1)ethan-1-al; (G) benzo[c][1,2]oxathiin S,S-dioxide.

The majority of DBT biodesulfurization bacteria, such as *Rhodococcus* sp. IGTS8, *Rhodococcus erythropolis* SHT87 [15], *Mycobacterium* sp. ZD-19 [16], *Pseudomonas stutzeri* TCE3 [17] and *Rhodococcus erythropolis* LSSE8-1 [18] seem to be incapable of BT desulphurization despite the similarity in their structures. Only a few bacteria harboring *dsz*ABC or their homologs (Table S1 in File S1), such as *Paenibacillus* sp. strain A11-2 [19,20], *Mycobacterium goodii* X7B [21], *Gordonia alkanivorans* RIPI90A [22,23] are capable of the biodesulfurization of both DBT and BT. However, the participation of DszA, DszB and DszC in the metabolism of BT has not yet been confirmed. *Gordonia* sp. 213E (NCIMB 40816) [24] and *Sinorhizobium* sp. KT55 [25] are specific BT biodesulfurization bacteria, which are capable of extracting sulfur from BT without breaking carbon-carbon bonds. Based on the characterization of metabolites produced during the BT biodesulfurization process by strain 213E and strain KT55, two types of BT biodesulfurization pathways have been proposed (Figure 1): (a) benzothiophene → benzothiophene S-oxide (BTO) → benzothiophene S,S-dioxide (BTO_2_) → benzo[c][l, 2]oxathiin S-oxide (BcOTO) → 2-(2’-hydroxypheny1)ethan-1-al [24]; (b) benzothiophene → benzothiophene S-oxide (BTO) → benzothiophene S,S-dioxide (BTO_2_) → benzo[c][l, 2]oxathiin S-oxide (BcOTO) → o-hydroxystyrene [25]. Metabolites of BTO_2_ and BcOTO were detected in these desulfurization pathways although their final desulfurization products are different. Some bacteria that desulfurize BT via these two routes have been isolated and characterized, such as *Paenibacillus* sp. A11-2, *Mycobacterium goodii* X7B could desulfurize BT to yield o-hydroxystyrene [20,21].

These previous studies have improved our understanding of the BT biodesulfurization mechanism, although the biochemical and genetic bases of the BT biodesulfurization pathway remain to be elucidated. *G. terrae* strain C-6 desulfurizes BT to yield a phenolic compound as the final product. It is hypothesized that this strain contains enzymes that are similar in function to those involved in DBT biodesulfurization, for that the biodesulfurization pathway and metabolites are so analogous to those of DBT biodesulfurization. Based on this hypothesis, we compared the transcriptomic profiles of strain C-6 cultured with BT or sodium sulfate as the sole source of sulfur. Among the significantly up-regulated genes, the *bds* (BT biodesulfurization) operon involved in the BT biodesulfurization pathway was identified. This information improves our knowledge of the mechanisms of aromatic thiophene metabolism.

## Results and Discussion

### Profiling of cell growth, BT utilization, and product yield of strain C-6 cultured with BT as sole source of sulfur

**Figure 2:**
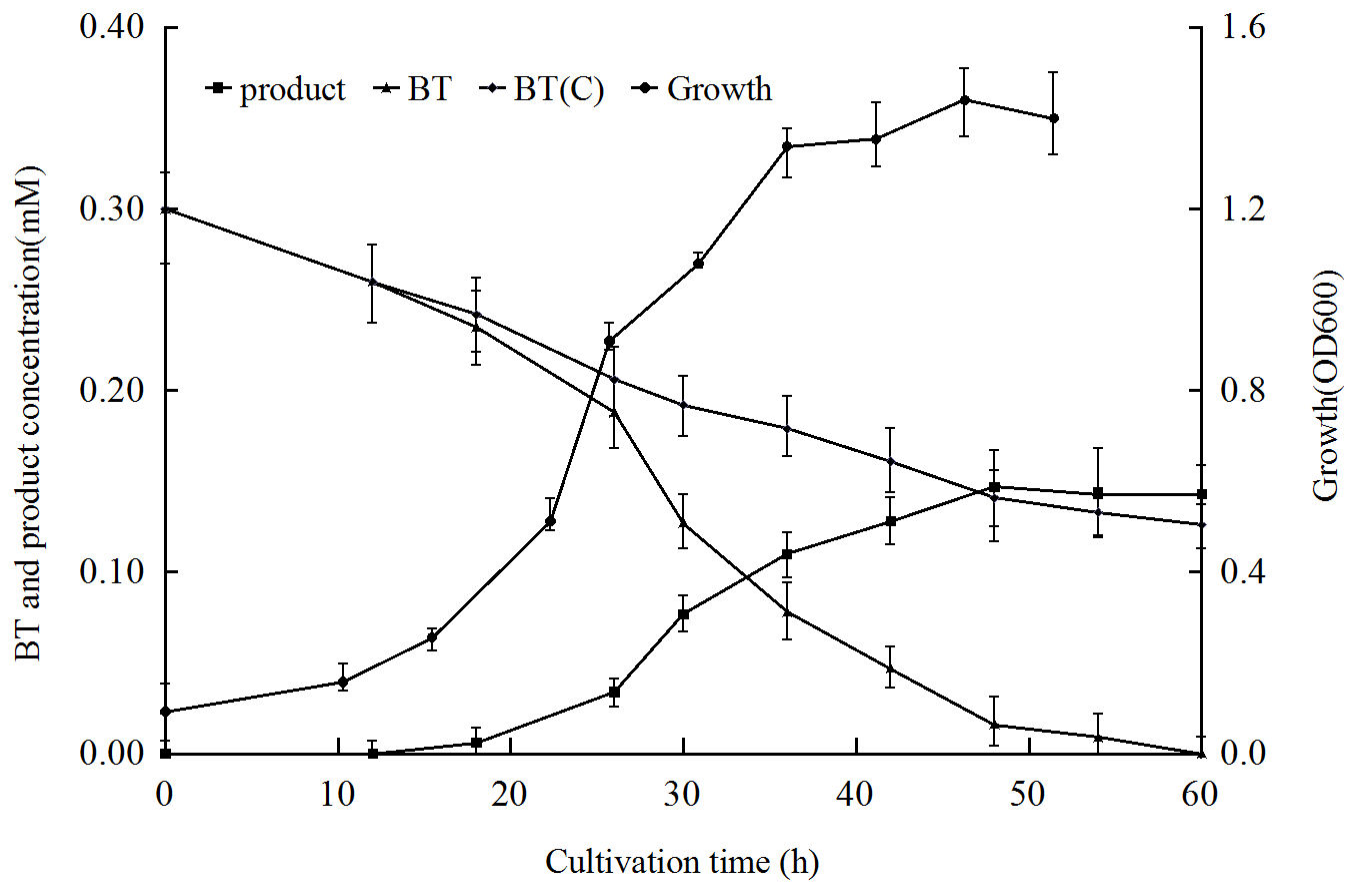
Biodesulfurization of BT during growth of *G. terrae* strain C-6. Strain C-6 was cultured in BSM_S-_ medium with 0.3mM BT as the sole source of sulfur. Black triangles, BT in the culture inoculating with strain C-6; black diamonds, BT in the culture without inoculating with strain C-6; black circles, bacterial growth; black squares, final product of BT biodesulfurization.

When strain C-6 was cultured in the BSM_S-_ medium containing BT as the sole sulfur source, maximum growth was achieved at 48 h, and the turbidity at 600nm at this time point was 1.36. No growth was observed without the addition of an alternative carbon source. These results indicate that this strain utilized BT as a sulfur source but not as sole carbon source. The Gibbs' assay showed that a phenolic compound was accumulated in the medium during the culture process, indicating that strain C-6 desulfurized BT to yield HPEal or o-hydroxystyrene according to the reported pathway [24,25]. The maximum phenolic compound formation, close to 50% of the initial BT concentration, was obtained after cultivation for 48 h. This indicated that the consumption of BT was more rapid than the production of the phenolic compound during the culture process (Figure 2). It can be speculated that this phenomenon is caused by the sublimation of BT, which is a sublimate organic compound at room temperature, in the culture process. This hypothesis was confirmed by loss of BT in a control experiment without inoculating strain C-6 in the culture (Figure 2).

### Global gene expression patterns of strain C-6 cultured with different sources of sulfur

**Figure 3:**
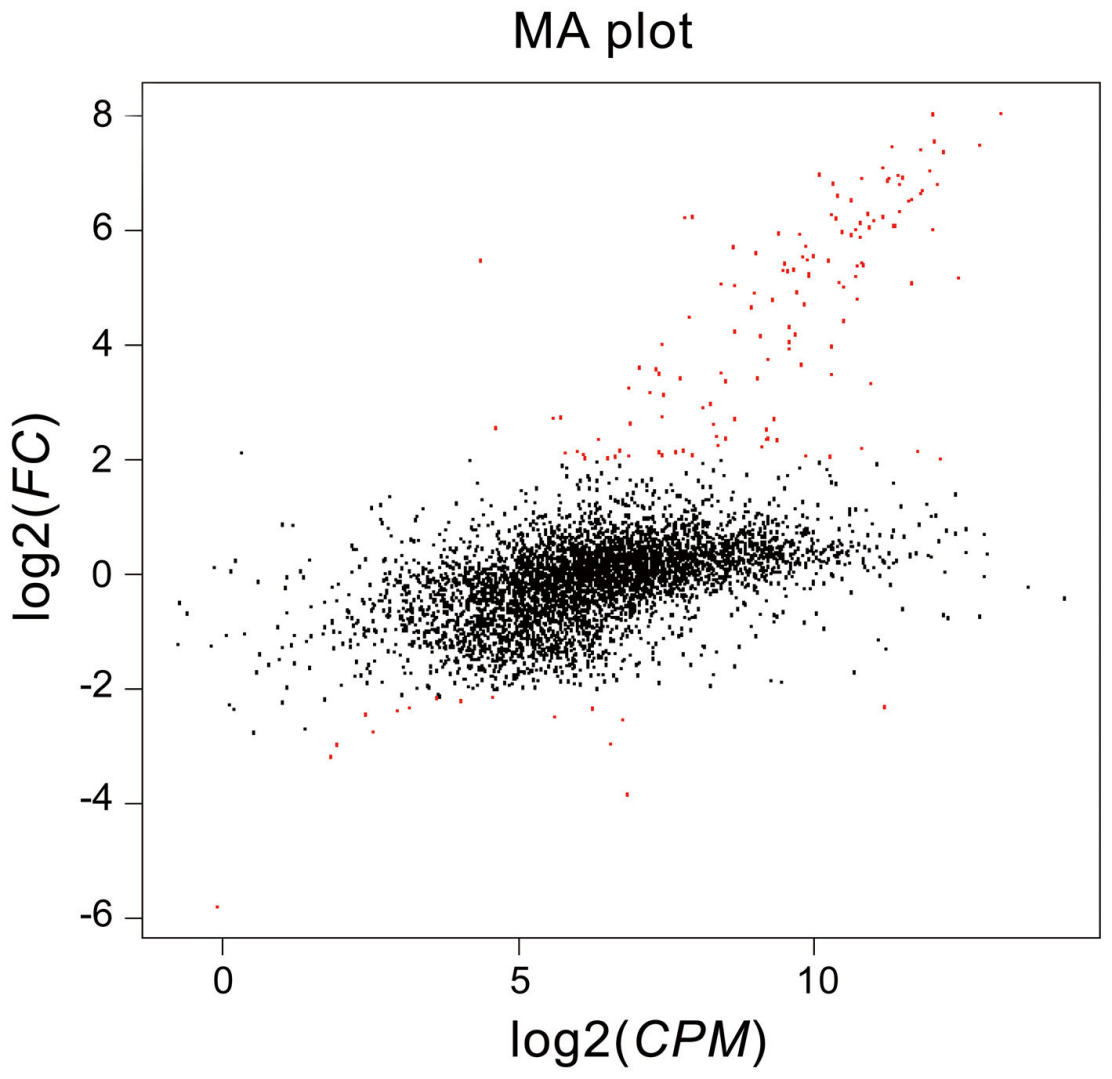
Differentially expressed genes of strain C-6 cultured with different sulfur sources. Red dots indicate differentially expressed genes. Black-colored dots were not considered as significantly differentially expressed. The Y-axis shows the fold-change values between BT and sodium sulfate as sulfur sourcebased on a log_2_ scale. The X-axis shows the average count of reads per million reads based on a log_2_ scale.

Most organisms cannot convert sulfur directly into biomass, especially the sulfur contained in heterocycles such as BT and DBT. It must be converted initially into SO_x_^2-^ by a series of enzymes involved in the aromatic thiophene biodesulfurization pathway, and then converted subsequently into biomass by another group enzymes involved in sulfur cycle, such as sulfite reductase [26,27]. In order to clarify the BT desulfurization mechanism of strain C-6, we investigated the differential expression of genes in strain C-6 cultured with BT or sodium sulfate as the sole source of sulfur. In this case, the up-regulated genes would be mainly related to sulfate limitation and/or induced by BT as part of BT desulfurization. To obtain an overview of strain C-6 gene expression profiles associated with growth on different sources of sulfur, cDNA samples were prepared and sequenced on anIllumina HiSeq 2000 platform. A total of 2,158,985,178 and 2,239,588,440 bases from 13,327,069 and 13,824,620 read pairs with a mean read length 81 bp were obtained from bacterial samples cultured with BT and sodium sulfate, respectively. These raw data were assembled into 3,157 contigs, and 4,800 unigenes were finally generated. Among these, 3,182 proteins had homologs in the COG database. With the exception of R, S categories of COG which are “function unknown” or “general function prediction only”, others were related to the normal physiological metabolism of cells, such as regulation, transport and cell processing (Figure S1 in File S1). In comparison with sodium sulfate as the sulfur source, the expression levels of 151 genes were deferent when BT was used as the sulfur source. Among these genes, 135 were up-regulated and 16 were down-regulated (Figure 3). Most of the significantly up-regulated genes (fold change more than 20) were shown to encode FMNH_2_-dependent monooxygenase, alkanesulfonate monooxygenase, desulfinase and transport related proteins, which are similar in function to enzymes involved DBT biodesulfurization of *Rhodococcus* sp. strain IGTS8 [8,9]. On this basis, 37 possible BT biodesulfurization related genes were selected and further investigated by RT-qPCR. All these genes were reconfirmed to be up-regulated in accordance with the RNA-seq result.

### *In silico* analysis of BT desulfurization pathway genes

Both types of proposed BT biodesulfurization pathways are initiated by the activity of a monooxygenase, which catalyzes the reaction BT →BTO →BTO_2_ (Figure 1) [20,24,25]. Five of the significantly up-regulated genes (Table 1) are predicted to encode FMNH2-dependent monooxygenases, suggesting that the catalytic mechanism is analogous to that of DBT monooxygenase (DszC) [28]. Five additional genes were predicted to encode alkanesulfonate monooxygenases, which contribute to the conversion of BTO_2_ to benzo[e][1,2]oxathiin S,S-dioxide (Table 1). A desulfinase encoding gene was also identified among the significantly up-regulated genes. Its product catalyzes the last step of the 4S desulfurization pathway, which involves the hydrolysis of sulfinate or sulfonate from benzene sulfinate or benzene sulfonate [29]. In addition to these genes proposed to be involved in the 4S desulfurization pathway, NAD(P)H-dependent flavin reductase encoding gene was also identified among the significantly up-regulated genes. It provides flavin-dependent monooxygenase with reduced flavin [28]. As a general rule, genes involved in the same metabolic pathway usually exist in the form of an operon on the chromosome or plasmid. Based on this hypothesis, we sequenced the genome of strain C-6[30]. The genetic map of chromosome reveals an operon, designated *bds*ABC (accession number:KC831580), containing only a desulfinase gene (Gene ID GTC6_01605, designated *bds*A), a FMNH_2_-dependent monooxygenase gene (Gene ID GTC6_01600, designated *bds*B) and an alkanesulfonate monooxygenase gene (Gene ID GTC6_01595, designated *bds*C), which encode enzymes functionally analogous to DszB, DszC and DszA respectively. Other up-regulated genes are dispersed throughout the chromosome of strain C-6. As the most likely operon involved in BT desulfurization, the *bds*ABC operon was selected for the subsequential analysis of desulfurization activity although enzymes encoded by this operon show low homology with that involved in the "4S" desulfurization pathway of DBT (Table S1 in File S1). Another group of significantly up-regulated genes was found to be related to the transmembrane transport of molecules, which maybe involved in the transport of BT and its metabolites [29].

**Table 1:** Significantly up-regulated genes of strain C-6 cultured with different sulfur source.

**Gene ID**	**Description**	**Expression ratio (BT/sodium sulfate)**		
		**fold by RT-qPCR**		**fold by RNA-seq**
**Group Ⅰ**				
GTC6_00280	alkanesulfonate monooxygenase [EC:1.14.14.5]	27±2	111	
GTC6_04560	NADPH-dependent FMN reductase	23±1	22	
GTC6_13080	putative FMNH2-dependent monooxygenase	11±3	43	
GTC6_15154	putative FMNH2-dependent monooxygenase	27±1	75	
GTC6_15109	alkanesulfonate monooxygenase (EC:1.14.14.5)	21±1	65	
GTC6_01595	alkanesulfonate monooxygenase [EC:1.14.14.5]	29±2	92	
GTC6_01600	putative FMNH2-dependent monooxygenase	42±1	169	
GTC6_01605	2'-hydroxybiphenyl-2-sulfinate desulfinase [EC:3.13.1.3]	36±1	125	
GTC6_06509	putative FMNH2-dependent monooxygenase	21±3	104	
GTC6_06474	putative FMNH2-dependent monooxygenase	38±1	72	
GTC6_06469	alkanesulfonate monooxygenase (EC:1.14.14.5)	29±1	111	
GTC6_06464	alkanesulfonate monooxygenase (EC:1.14.14.5)	49±2	121	
Group II				
GTC6_00275	sulfonate/nitrate/taurine transport system substrate-binding protein	30±1	100	
GTC6_00260	sulfonate/nitrate/taurine transport system ATP-binding protein	52±2	126	
GTC6_00255	sulfonate/nitrate/taurine transport system permease protein	45±1	70	
GTC6_10396	ABC transporter	14±1	34	
GTC6_10391	binding-protein-dependent transport systems inner membrane component	10±2	30	
GTC6_10386	binding-protein-dependent transport systems inner membrane component	13±1	30	
GTC6_11646	putative ABC transporter substrate binding protein	22±1	68	
GTC6_11651	ABC peptide transporter, permease component	18±1	47	
GTC6_11656	ABC peptide transporter, permease component	12±1	44	
GTC6_11661	ABC peptide transporter, ATP-binding component	32±1	78	
GTC6_13085	putative peptide ABC transporter substrate binding protein	25±3	42	
GTC6_13090	ABC transporter inner membrane protein	10±1	37	
GTC6_13095	putative ABC transporter	11±1	39	
GTC6_13100	ABC transporter-like protein	37±1	59	
GTC6_15124	sulfonate/nitrate/taurine transport system permease protein	20±2	63	
GTC6_15119	sulfonate/nitrate/taurine transport system ATP-binding protein	23±2	48	
GTC6_15114	sulfonate/nitrate/taurine transport system substrate-binding protein	34±1	61	
GTC6_01585	ABC transporter substrate-binding protein	51±1	188	
GTC6_01590	hypothetical protein	54±3	176	
GTC6_01610	sulfonate/nitrate/taurine transport system ATP-binding protein	34±1	112	
GTC6_01615	sulfonate/nitrate/taurine transport system permease protein	50±1	120	
GTC6_01620	putative ABC transporter substrate-binding protein	30±4	165	
GTC6_15606	sulfonate/nitrate/taurine transport system ATP-binding protein	8±1	25	
GTC6_15611	sulfonate/nitrate/taurine transport system substrate-binding protein	12±1	45	
GTC6_15616	sulfonate/nitrate/taurine transport system permease protein	12±2	28	

### Involvement of the *bds*ABC operon in BT biodesulfurization

**Figure 4:**
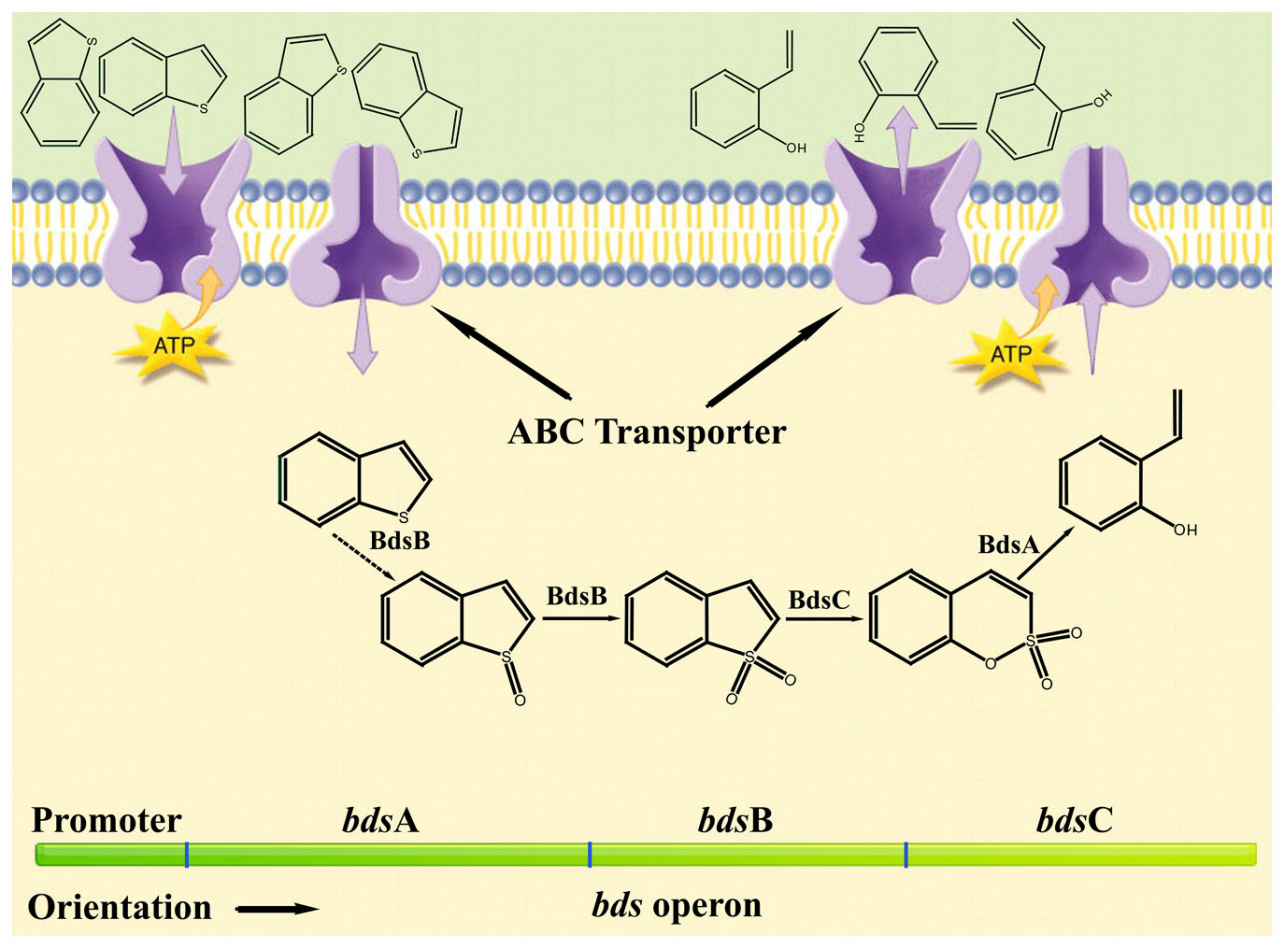
The proposed metabolism of BT for *G. terrae* strain C-6.

To verify the putative roles of the *bds*ABC operon in the BT biodesulfurization pathway, we cloned and overexpressed this operon in *E. coli* Rosetta (DE3) with the expression vector pET28a (+). As expected, a phenolic compound was accumulated (indicated by the positive result in the Gibbs′ assay) when BT was incubated with cell extracts of *E. coli* Rosetta (DE3) overexpressing pET28a-*bds*ABC. NADH, FMN and *dsz*D, a NAD(P)H-dependent flavin reductase involved in the DBT biodesulfurization pathway [28], were essential for the activity of these cell extracts, indicating that some of these enzymes involved in the BT biodesulfurization pathway are also flavin-dependent. In order to identify the structure of this phenolic compound and intermediate metabolites, the reaction mixture was extracted directly with ethyl acetate after incubation for 24h at 30°C. Approximately 95% volume of the ethyl acetate extracts were volatilized at room temperature for concentrating BT biodesulfurization metabolites. The remaining extracts were analyzed by GC-MS. Compared with the control experiment in which cell extracts of *E. coli* Rosetta (DE3)- pET28a-*bds*ABC were replaced with that of *E. coli* Rosetta (DE3), no other different peaks were identified by the GC profiles, with the exception of the peak with retention time of 3.67 min. MS spectra of this peak are identical to that of o-hydroxystyrene (Figure S2 in File S1). Nonetheless, the BT biodesulfurization pathway, which is analogous to the 4S pathway of DBT, is becoming clearer based on the genetic analysis of BT metabolism, the final metabolite of BT and the results reported previously (Figure 4) [20,24,25].

## Conclusion

*G. terrae* strain C-6 desulfurizes BT to yield o-hydroxystyrene by mechanism analogous to the 4S biodesulfurization pathway. Transcriptomic profiles of strain C-6 cultured with BT or sodium sulfate as the sole source of sulfur indicated that three genes were involved in the BT biodesulfurization. These genes were located in the same operon. Cell extracts of *E. coli* Rosetta (DE3) overexpressing pET28a-*bds*ABC also converted BT to o-hydroxystyrene, which supported the hypothesis that the *bds*ABC operon is involved in BT biodesulfurization.

## Materials and Methods

### Chemicals

BT, BTO_2_ and FMN were purchased from Sigma-Aldrich (Missouri, USA). Kanamycin and NADH were purchased from Amresco (Ohio, USA). Isopropyl-β-D-1-thiogalactopyranoside (IPTG) was purchased from Merck (New Jersey, USA). Polymerase chain reaction (PCR) primers were synthetized by Sangon (Shanghai, China). *Pfu* DNA polymerase was obtained from Promega (Wisconsin, USA). T4 DNA ligase and restriction endonucleases were obtained from Fermantas (Maryland, USA). All other reagents were of chromatography grade and were obtained from various commercial sources.

### Bacterial strains and cultivation conditions

*Gordonia terrae* C-6 is capable of desulfurizing BT and its derivatives but not DBT and its derivatives (Table S2 in File S1). Strain C-6 and *E. coli* Rosetta (DE3) overexpressing pET28a-*bds*ABC were incubated in 250 ml Erlenmeyer flask (liquid volume, 100 ml) with basal salts medium (BSM_S-_) lacking a sulfur source at 30°C with shaking (180 r/min), and with 0.3mM BT or sodium sulfate as the sole source of sulfur. BSM_S-_contained (per liter): 4.00 g glycerol, 2.44 g KH_2_PO_4_, 14.04 g Na_2_HPO_4_·12H_2_O, 2.00 g NH_4_Cl, 0.40 g MgCl_2_·6H_2_O, 0.01 g FeCl_3_ and 0.02 g CaCl_2_.

### Substrate and product analysis

Quantitative analysis of BT was performed by HPLC (Agilent 1100 series, California, USA) fitted with a ZORBAX SB-C18 column (4.6 mm i.d. × 250 mm length). The mobile phase consisting of 80% methanol and 20% water was pumped at a flow rate of 1 ml/min. The absorbance of BT was measured at 245 nm. Final biodesulfurization product of BT was quantified by Gibbs′ assay according to previously described methods [31]. Qualitative analysis of BT biodesulfurization products was performed according to previously described methods [25]. Before analysis, BT and its metabolites in the aqueous phase were extracted with an equal volume of ethyl acetate. The ethyl acetate layer was centrifuged (1,600 ×*g*, 10min), and the supernatant was analyzed by gas chromatography (GC) or gas chromatography-mass spectrometry (GC-MS) according to previously reported methods[25].

### DNA and RNA extraction

Strain C-6was cultured with BT or sodium sulfate as the sole sulfur source. Cells in culture were harvested by centrifugation (1,200 ×*g*, 10min) at the end of the logarithmic growth phase. Following incubation of cells at 37°C for 10 min with 125 μg/ml lysostaphin (AMBI, New York, USA), genomic DNA extraction was carried out with a bacterial genomic DNA purification kit (EdgeBio, Maryland, USA) according to the manufacturer’s instructions. Cells were resuspended with TRIzol and disrupted with Mini-Bead beater (Biospec, California, USA).Total RNA was isolated according to the manufacturer’s instructions of TRIzol-chloroform extraction (Invitrogen, California, USA). Genomic DNA was removed with Turbo DNase. Ribosomal RNA (rRNA) was removed with the Ribo-Zero Magnetic kit (G+/G-Bacteria) (Epicentre, Wisconsin, USA).

### RNA sequencing and data analysis

The cDNA library was constructed with extracted mRNA by the TruseqTM RNA sample prep kit (Illumina, California, USA). Sequencing was carried out on the Illumina HiSeq 2000 platformat Majorbio Bio-Pharm Technology Co., Ltd., Shanghai, China.

The RNA-seq data from both of the samples were assembled by Trinity [32], and unigenes were predicted with Metagene (prokaryotic gene identification from environmental genome shotgun sequences).The functions of unigenes were annotated by searching against the non-redundant protein database with Blastp with E values less than 1.0×10^−5^. The clusters of orthologous groups (COG) functional categories were assigned with the STRING database (String v9.1). Reads from each sample were mapped to unigenes by BowTie software [33]. The expression of each unigene was calculated with the numbers of reads mapping to it. Differences in gene expression were analyzed by the R package, edgeR [34]. Genes with Q value <0.1 and fold-change 2were assigned as differentially expressed. Sequences were deposited in the NCBI SRA database under the accession no. SRA092218.

### Cloning and expression of bds operon

The *bds* operon was amplified from the genomic DNA of strain C-6 by PCR with the following primers: 5’-ACACCATGGCTGAGGACGAAACCCCGATGACC-3’ and 5’-GTGTGGAAGCTTGTTCTGTGGCAGGGGCTTCAG-3’. Sequence was deposited in the Genbank under the accession no. KC831580.The amplified product was ligated into the *Nco*I and *Hind*III sites of pET28a (+) to yield pET28a-*bds*ABC. This recombinant plasmid was introduced into *E. coli* Rosetta (DE3) by electroporation with a Micropulser electroporator (Bio-Rad, California, USA) as described previously to yield *E. coli* Rosetta (DE3) overexpressing pET28a-*bds*ABC [35].

### Enzymatic assays

*E. coli* Rosetta (DE3) overexpressing pET28a-*bds*ABC was cultured in BSM_S-_ with 0.3mM sodium sulfate as the sole source of sulfur (34 mg/L Kanamycin ) at 37°C to an optical density (OD) of 0.8 at 600 nm. The culture was then induced with 0.06 mM IPTG to express enzymes encoded by *bds*ABC. In order to avoid the formation of inclusion bodies, the cultivation temperature was reduced to 16°C. The cells were harvested by centrifugation (12,000×*g*, 5 min) at 4°C and washed with 0.85% (w/v) sodium chloride solution after incubation for 48h. Cells extracts were prepared as previously described [28]. Enzymatic assays were performed with 0.5ml cell extract supernatants, 0.5mM BT, 20 μM FMN, 1mM NADH, 10nM DszD purified from *E. coli* (DE3)-pET28a-*dsz*D in100 mM pH 7.2 KPi buffer (total of 5 ml) at 30°C. Metabolites of this reaction system were analyzed with GC-MS according to previously reported methods [25].

### Quantitative real-time PCR (RT-qPCR) verification

37 genes proposed involving BT biodesulfurization were chosen for the confirmation of RNA-seq data by qPCR with the SYBR Premix Ex Taq kit (Takara, Japan) according to the manufacturer’s instructions with a real-time thermal cycler (Bio-Rad, Hercules, CA). Templates used for qPCR were cDNAs that was inverse transcribed from the extracted mRNA using M-MLV Reverse Transcriptase (Takara, Japan) with random primers. The primers used for RT-qPCR detection of selected genes are listed in Table S3 in File S1. The relative gene expression data were analyzed using the 2^-ΔΔCt^method as described previously [36]. The results were analyzed using a one-way analysis of variance (ANOVA) statistical test. All quantitative PCR were repeated in three biological and three technical replications.

## Supporting Information

File S1 **Figure S1, Histogram of clusters of orthologous groups (COG) classification.** A, RNA processing and modification; B, Chromatin structure and dynamics; C, Energy production and conversion; D, Cell cycle control, cell division, chromosome partitioning; E, Amino acid transport and metabolism; F, Nucleotide transport and metabolism; G, Carbohydrate transport and metabolism; H, Coenzyme transport and metabolism; I, Lipid transport and metabolism; J, Translation, ribosomal structure and biogenesis; K, Transcription; L, Replication, recombination and repair; M, Cell wall/membrane/envelope biogenesis; N, Cell motility; O, Posttranslational modification, protein turnover, chaperones; P, Inorganic ion transport and metabolism; Q, Secondary metabolites biosynthesis, transport and catabolism; R, General function prediction only; S, Function unknown; T, Signal transduction mechanisms; U, Intracellular trafficking, secretion, and vesicular transport; V, Defense mechanisms; W; Extracellular structures; Y, Nuclear structure; Z, Cytoskeleton. **Figure S2, MS spectra of the phenolic compound produced by cell extracts of *E. coli* Rosetta (DE3) conceived with pET28a-*bds*ABC during BT biodesulfurization**. Metabolites of BT in the cell extracts reaction system was extracted directly with ethyl acetate without adjusting its pH to 2.0, and analyzed by GC-MS according to the previously described method [25]. o-hydroxystyrene was identified by MS spectra corresponding to the peak with retention time of 3.67 in the GC profile. The molecular weight of o-hydroxystyrene decreased by 1 (the hydroxyl of o-hydroxystyrene exists in the form of negative ions) due to the extracting procedure. **Table S1, The amino acid homology of BT/DBT biodesulfurization enzymes. Table S2, in File S1 Sulfur resource specificity of *Gordonia terrae* C-6. Table S3, in File S1 Primers used for RT-qPCR**.
(DOCX)
