# Supplementary material for: Genetic Analysis of Benzothiophene Biodesulfurization Pathway of Gordonia terrae Strain C-6
Source: PLoS One. 2013 Dec 19;8(12):e84386. doi: 10.1371/journal.pone.0084386 (PMC3868597; doi:10.1371/journal.pone.0084386)
Supplement: File S1 — Figure S1, Histogram of clusters of orthologous groups (COG) classification. A, RNA processing and modification; B, Chromatin structure and dynamics; C, Energy production and conversion; D, Cell cycle control, cell division, chromosome partitioning; E, Amino acid transport and metabolism; F, Nucleotide transport and metabolism; G, Carbohydrate transport and metabolism; H, Coenzyme transport and metabolism; I, Lipid transport and metabolism; J, Translation, ribosomal structure and biogenesis; K, Transcription; L, Replication, recombination and repair; M, Cell wall/membrane/envelope biogenesis; N, Cell motility; O, Posttranslational modification, protein turnover, chaperones; P, Inorganic ion transport and metabolism; Q, Secondary metabolites biosynthesis, transport and catabolism; R, General function prediction only; S, Function unknown; T, Signal transduction mechanisms; U, Intracellular trafficking, secretion, and vesicular transport; V, Defense mechanisms; W; Extracellular structures; Y, Nuclear structure; Z, Cytoskeleton. Figure S2, MS spectra of the phenolic compound produced by cell extracts of E. coli Rosetta (DE3) conceived with pET28a-bdsABC during BT biodesulfurization. Metabolites of BT in the cell extracts reaction system was extracted directly with ethyl acetate without adjusting its pH to 2.0, and analyzed by GC-MS according to the previously described method [25]. o-hydroxystyrene was identified by MS spectra corresponding to the peak with retention time of 3.67 in the GC profile. The molecular weight of o-hydroxystyrene decreased by 1 (the hydroxyl of o-hydroxystyrene exists in the form of negative ions) due to the extracting procedure. Table S1, The amino acid homology of BT/DBT biodesulfurization enzymes. Table S2, in File S1 Sulfur resource specificity of Gordonia terrae C-6. Table S3, in File S1 Primers used for RT-qPCR. (DOCX) [file pone.0084386.s001.docx]

**Table S1 The amino acid homology of BT/DBT biodesulfurization enzymes.**

|  | | RIPI90A^PR^ | | | A11-2^PR^ | | | X7B^PR^ | | | IGTS8^PP^ | | |
| --- | --- | --- | --- | --- | --- | --- | --- | --- | --- | --- | --- | --- | --- |
|  |  | DszA | DszB | DszC | TdsA | TdsB | TdsC | DszA | DszB | DszC | DszA | DszB | DszC |
| C-6^PR^ | BdsC | 12.4^Id^ | 13.3^Id^ | 9.6^Id^ | 12.5^Id^ | 11.4^Id^ | 12.2^Id^ | 13.0^Id^ | 11.1^Id^ | 10.3^Id^ | 11.7^Id^ | 10.1^Id^ | 9.8^Id^ |
|  | BdsA | 10.6^Id^ | 22.9^Id^ | 10.4^Id^ | 9.3^Id^ | 16.6^Id^ | 11.1^Id^ | 10.3^Id^ | 16.6^Id^ | 11.1^Id^ | 10.3^Id^ | 23.2^Id^ | 9.1^Id^ |
|  | BdsB | 13.7^Id^ | 14.3^Id^ | 12.2^Id^ | 13.9^Id^ | 14.8^Id^ | 9.6^Id^ | 13.7^Id^ | 13.5^Id^ | 11.3^Id^ | 14.4^Id^ | 13.0^Id^ | 12.4^Id^ |
| RIPI90A^PR^ | DszA |  |  |  | 59.8^Id^ |  |  | 75.0^Id^ |  |  | 87.1^Id^ |  |  |
|  | DszB |  |  |  |  | 50.0^Id^ |  |  | 62.4^Id^ |  |  | 86.9^Id^ |  |
|  | DszC |  |  |  |  |  | 50.6^Id^ |  |  | 72.4^Id^ |  |  | 90.4^Id^ |
| A11-2^PR^ | TdsA |  |  |  |  |  |  | 61.9^Id^ |  |  | 61.9^Id^ |  |  |
|  | TdsB |  |  |  |  |  |  |  | 52.2^Id^ |  |  | 51.1^Id^ |  |
|  | TdsC |  |  |  |  |  |  |  |  | 50.2^Id^ |  |  | 49.5^Id^ |
| X7B^PR^ | DszA |  |  |  |  |  |  |  |  |  | 77.3^Id^ |  |  |
|  | DszB |  |  |  |  |  |  |  |  |  |  | 63.2^Id^ |  |
|  | DszC |  |  |  |  |  |  |  |  |  |  |  | 71.2^Id^ |

Note: ^PR^, Protein Resource; ^Id^, Identities percentage; C-6, *Gordonia terrae* C-6; RIPI90A, *Gordonia alkanivorans* RIPI90A; A11-2, *Paenibacillus* sp. strain A11-2; X7B, *Mycobacterium goodii* X7B; IGTS8, *Rhodococcus* sp. IGTS8. DszA and TdsA, dibenzothiophene-5,5-dioxide monooxygenase; DszB and TdsB, 2-hydroxybiphenyl-2-sulfinate sulfinolyase; DszC and TdsC, dibenzothiophene monooxygenase; BdsA, desulfinase; BdsB, FMNH2-dependent monooxygenase; BdsC, alkanesulfonate monooxygenase.

**Table S2 Sulfur resource specificity of *Gordonia terrae* C-6.**

| **Sulfur resource** | ***OD*_600_** | **Gibbs′ assay** |
| --- | --- | --- |
| control | 0.00 | − |
| sodium sulfate | 0.92 | − |
| yeast extract | 1.26 | − |
| casein hydrolysate | 1.17 | − |
| dimethyl sulfoxide | 1.50 | − |
| benzothiophene | 1.44 | + |
| 2-methyl-benzothiophene | 0.96 | + |
| 3-methyl-benzothiophene | 0.82 | + |
| 5-methyl-benzothiophene | 1.33 | + |
| 2-carboxyl-benzothiophene | 0.23 | − |
| dibenzothiophene | 0.15 | − |
| 1-methyl- dibenzothiophene | 0.15 | − |
| 2-methyl- dibenzothiophene | 0.13 | − |
| 3-methyl- dibenzothiophene | 0.16 | − |
| 4-methyl- dibenzothiophene | 0.19 | − |

Note: +, indicates positive result in the Gibbs′ assay; −, indicates negative result in the Gibbs′ assay;

**Table S3 Primers used for RT-qPCR.**

| **Gene ID** | **Forward primer (5′→3′)** | **Reverse primer(5′→3′)** |
| --- | --- | --- |
| GTC6_00280 | CCTCGAAGGGCTGGAACTGC | CGGTCCCGTCCGCAGATCTAC |
| GTC6_00275 | GTCTTCGATGCTGCCCTGTT | GTGCTCGAAGGGCTCCTACAT |
| GTC6_00260 | CGTCGTGCGTCACCAGCAAG | CCTGGCCGTCCACGCTCTC |
| GTC6_00255 | TCGTTCATCAGAAAGCCCAATC | AGGCGGGATGGGAACTGAT |
| GTC6_04560 | CAGACGGCGTGCTCATCG | GGGAAGAGCGGCGACCTG |
| GTC6_10396 | TGGCGGGCCTGGATGTCTC | CAGGGCAGTTATCCGCACCAG |
| GTC6_10391 | AGATGAGGCGGCTCAAGATG | CACGACTATCCCGTCATCCAG |
| GTC6_10386 | GCCGGGAGGATGAGGTGA | TACGGCGTTCGGCAACAC |
| GTC6_11646 | GTCGGCATCAATGTCACCAAT | CGTGAACTGAGCGAAACCGT |
| GTC6_11651 | GGCAGCATCGGCGACCTC | GAGTGCGGGCAGCAGCAGAT |
| GTC6_11656 | GTTCCCGATCCCCGTTGTG | GCGTGATGCCGAGGATCAG |
| GTC6_11661 | GCGGACAGAAGCAGCGAGTG | CGGTGTGACGCACGGTGATC |
| GTC6_13080 | GGCGACGGCGAGAGGATG | CGCAGCAGCACCGCTTCAAC |
| GTC6_13085 | CCGAACGCCATCACCCTCAAC | TTGTGCGGGTTGGGACTGTCC |
| GTC6_13090 | ACGGCGATCTCGGCATCTC | GACGGTTTCGGCGATCACC |
| GTC6_13095 | GCTCGTGGTTCTCGCCCTGT | GGTCGCCGCCTCGATGTAGG |
| GTC6_13100 | AATCGCTGACCGCTCATGGT | GCCTTGCCGCTGAACGACT |
| GTC6_15154 | CGATTCCCGCCTGGATGAGC | CGCCTTCTTCGCCGACCTG |
| GTC6_15124 | CATCGCCGTCCTCGTGCTC | CACCGATGCCGAACCACAAG |
| GTC6_15119 | CAGCGGCAGTGGCAAGTCG | GGCGAGGGAGACCCGTTGTG |
| GTC6_15114 | CGCCTCCCTACAGGACCTCAC | GAACGCCGACAACGCATCT |
| GTC6_15109 | GCGCGAGACCTCGGAGGAG | GCCTTCGGCGAACCAGTGC |
| GTC6_01585 | GCCAGTTCCTCGGGATGCTG | GGTTTCACGCCACCGATCAC |
| GTC6_01590 | GTTGATGTGCGGCGGGATC | CCTGGGCCGACGACCTGAC |
| GTC6_01595 | GGCGGAATCGGTCGTGCTC | CACCGCAACTCGCACAGACG |
| GTC6_01600 | GGATGGATGTCGCCGTGTTCC | CCGTTGCCAAAGGCGTATG |
| GTC6_01605 | GCAGGTCCAGCCGCTCCTC | ATCGGTGCCGTCGGGATTT |
| GTC6_01610 | GCCGAAGGTGCCGTCCGACAG | ACCATCAGGCGCGGCGAGTTC |
| GTC6_01615 | TCGACAAGCAGATCCGCAGTG | CCGTTGTTCATCGCCTGGTTC |
| GTC6_01620 | CGGAAGGCGACGATGGTG | GGCGGGCAAGGGTGAGTAC |
| GTC6_06509 | TCGTGAGGCGGGCATCTTC | CCGCTGGCGTAGGACTGGT |
| GTC6_06474 | TCGACACCGACGCCCACTC | CCGAGGTGGTGGCGATCCT |
| GTC6_06469 | GAAGCCGCCCAGGATGAGGT | AGCCCAAGCCGCTCAACAC |
| GTC6_06464 | AGGTAGGTGTCGGCGTGCTT | CCACCGAGAGCCTCAAGTTCC |
| GTC6_15606 | GGTGAGCACGATGATCCGACT | GGAGTTCCTGGTGCTGGTGG |
| GTC6_15611 | GGTGTTCACGACCTCGGGAT | CCGATCTCAAGGGCAAGCG |
| GTC6_15616 | TTGACCACGCCACCGATTC | TGACCGCCGTTGGCCTACTT |


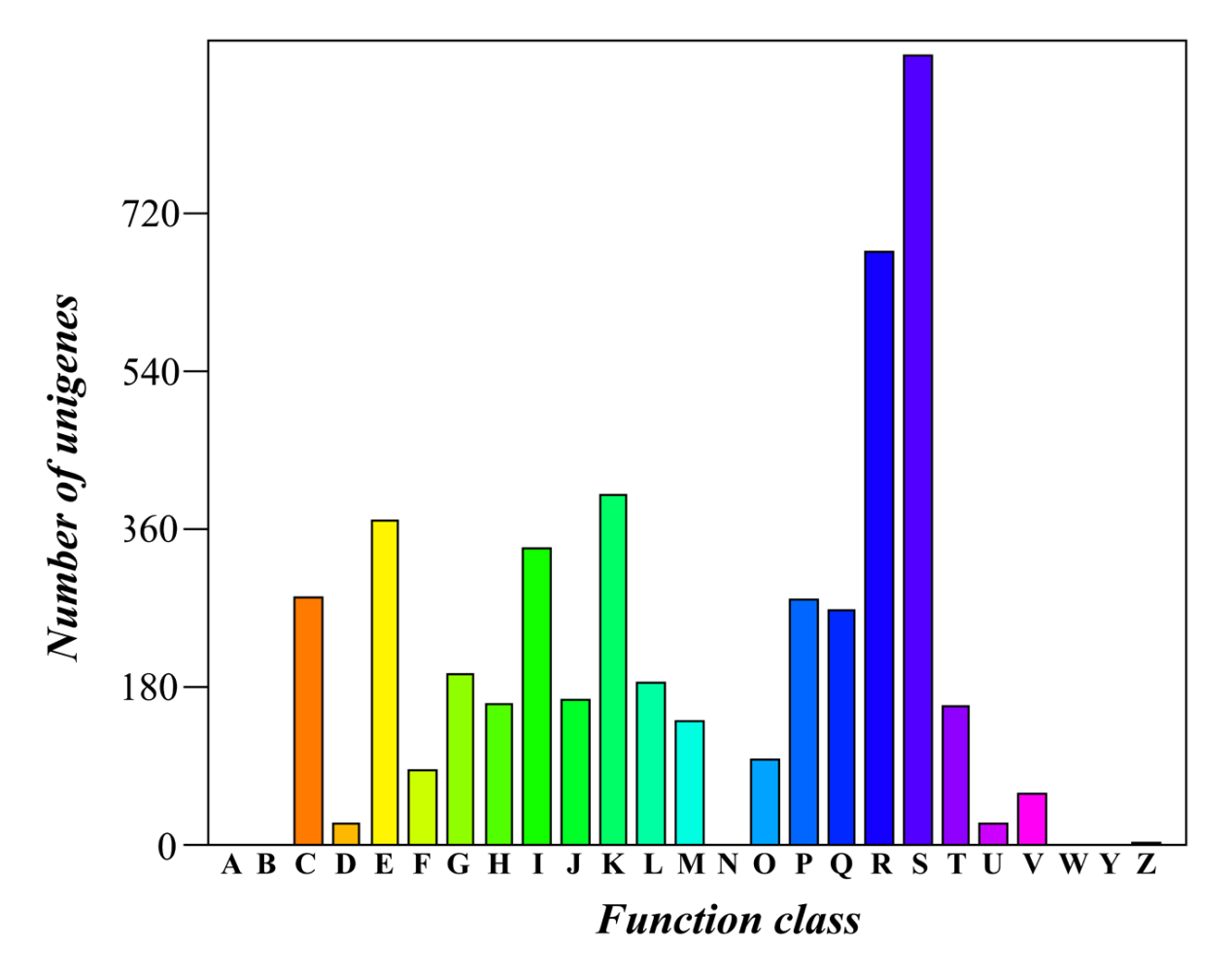


**Figure S1 Histogram of clusters of orthologous groups (COG) classification.**

A, RNA processing and modification; B, Chromatin structure and dynamics; C, Energy production and conversion; D, Cell cycle control, cell division, chromosome partitioning; E, Amino acid transport and metabolism; F, Nucleotide transport and metabolism; G, Carbohydrate transport and metabolism; H, Coenzyme transport and metabolism; I, Lipid transport and metabolism; J, Translation, ribosomal structure and biogenesis; K, Transcription; L, Replication, recombination and repair; M, Cell wall/membrane/envelope biogenesis; N, Cell motility; O, Posttranslational modification, protein turnover, chaperones; P, Inorganic ion transport and metabolism; Q, Secondary metabolites biosynthesis, transport and catabolism; R, General function prediction only; S, Function unknown; T, Signal transduction mechanisms; U, Intracellular trafficking, secretion, and vesicular transport; V, Defense mechanisms; W; Extracellular structures; Y, Nuclear structure; Z, Cytoskeleton.


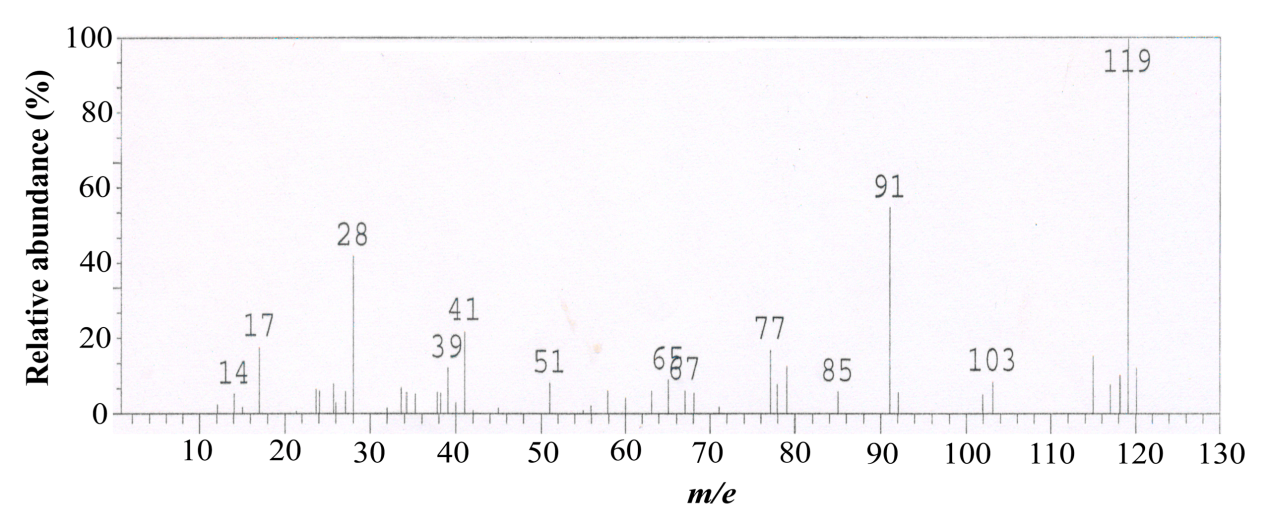


**Figure S2 MS spectra of the phenolic compound produced by cell extracts of *E. coli* Rosetta (DE3) conceived with pET28a-*bds*ABC during BT biodesulfurization.**

Metabolites of BT in the cell extracts reaction system was extracted directly with ethyl acetate without adjusting its pH to 2.0, and analyzed by GC-MS according to the previously described method [25]. *o*-hydroxystyrene was identified by MS spectra corresponding to the peak with retention time of 3.67 in the GC profile. The molecular weight of *o*-hydroxystyrene decreased by 1 (the hydroxyl of *o*-hydroxystyrene exists in the form of negative ions) due to the extracting procedure.
